# Optimization of Ohmic Contacts to p-GaAs Nanowires

**DOI:** 10.1186/s11671-019-3175-8

**Published:** 2019-11-14

**Authors:** Marcelo Rizzo Piton, Teemu Hakkarainen, Joonas Hilska, Eero Koivusalo, Donald Lupo, Helder Vinicius Avanço Galeti, Yara Galvão Gobato, Mircea Guina

**Affiliations:** 1 Optoelectronics Research Centre, Physics Unit, Tampere University, Tampere, Finland; 2 Physics Department, Federal University of São Carlos, São Carlos, Sao Paulo Brazil; 3 Electronics and Communications Engineering, Tampere University, Tampere, Finland; 4 Electrical Engineering Department, Federal University of São Carlos, São Carlos, Sao Paulo Brazil

**Keywords:** Nanowires, GaAs, p-type doping, Ohmic contacts

## Abstract

The performance of Ohmic contacts applied to semiconductor nanowires (NWs) is an important aspect for enabling their use in electronic or optoelectronic devices. Due to the small dimensions and specific surface orientation of NWs, the standard processing technology widely developed for planar heterostructures cannot be directly applied. Here, we report on the fabrication and optimization of Pt/Ti/Pt/Au Ohmic contacts for p-type GaAs nanowires grown by molecular beam epitaxy. The devices were characterized by current–voltage (IV) measurements. The linearity of the IV characteristics curves of individual nanowires was optimized by adjusting the layout of the contact metal layers, the surface treatment prior to metal evaporation, and post-processing thermal annealing. Our results reveal that the contact resistance is remarkably decreased when a Pt layer is deposited on the GaAs nanowire prior to the traditional Ti/Pt/Au multilayer layout used for p-type planar GaAs. These findings are explained by an improved quality of the metal-GaAs interface, which was evidenced by grazing incidence X-ray diffraction measurements in similar metallic thin films deposited on GaAs (110) substrates. In particular, we show that Ti exhibits low degree of crystallinity when deposited on GaAs (110) surface which directly affects the contact resistance of the NW devices. The deposition of a thin Pt layer on the NWs prior to Ti/Pt/Au results in a 95% decrease in the total electrical resistance of Be-doped GaAs NWs which is associated to the higher degree of crystallinity of Pt than Ti when deposited directly on GaAs (110).

## Introduction

An important step in the fabrication of semiconductor electronic and optoelectronic devices is to obtain high-quality and reliable Ohmic contacts at the metal-semiconductor interface. To this end, GaAs is an important and widely used material in technological applications such as laser devices, solar cells, and photodetectors. Therefore, the fabrication of Ohmic contact to p-type- and n-type-doped GaAs layers has been the target of a large number of investigations [1]. Generally speaking, the fabrication of Ohmic contacts to semiconductor materials include four steps: (1) removal of the surface native oxide, (2) passivation of the surface states at the semiconductor-metal interface, (3) deposition of metallic layers acting as the electrical contacts, and (4) thermal annealing [2, 3]. Starting from the selection of metals that provide low contact resistance and excellent thermal stability, Au-based alloys have been widely exploited, specifically for p-type GaAs in the form of Au/Zn/Au [1, 3, 4] and Ti/Pt/Au [1, 5] layers. On the other hand, semiconductor III–V nanowires (NWs) have emerged as newer class of promising nanoscale materials for application as LEDs [6, 7], solar cells [8, 9], and photodetectors [10], and these have triggered specific developments on device processing.

Majority of the technology reported in the literature for Ohmic contact fabrication was developed for GaAs (100) thin films and bulk structures, while the self-catalyzed growth of GaAs NWs yields (110) surface orientation at the sidewalls [11–15]. Different surface orientations exhibit different electronic states [16] which affect the interface properties and Schottky barrier heights [17–19]. In addition, the surface orientation may affect the crystallization dynamics of the deposited metal films. Good Ohmic contacts to p-GaAs NWs were previously reported [20–28] by using a variation of chemical treatments to remove the native oxide, surface passivation, and different metallic multilayers deposited on the NWs. Then, the use of Pt/Ti/Pt/Au electrical contacts to p-type GaAs thin films was reported to yield low Ohmic contact resistivity even for moderate dopant concentrations due to the low Schottky barrier height of Pt/GaAs [29]. In addition, Pt was found to be thermally stable with small reaction rates to GaAs for heat treatments with temperatures ranging from 300 to 500 °C [29–31]. Pt/Ti/Pt/Au metallic multilayer is the most frequently reported [20, 23, 25, 26, 28] Ohmic contact to p-GaAs NWs. However, a more comprehensive understanding of metal properties on Ohmic contact formation is the key to further improve the electrical contact quality on nanoscale devices. Due to the wide range of NW growth techniques and dopant concentrations in p-GaAs NWs recently reported, it would be unviable to make a comparison of the contact resistances, when they are available. Instead, a comprehensive investigation of different contact manufacturing routes on the same set of NW samples would be more suitable to exclude the effect of NW properties.

Here, we address the effects of different Pt- and Ti-based electrical contacts and surface treatments prior to metal evaporation on the overall current–voltage (IV) characteristics of self-catalyzed Be-doped GaAs NWs grown on Si substrates [11, 32]. We analyze the changes in the total resistance of the NW channels based on structural investigation by X-ray diffraction of Ti, Pt, and Pt/Ti thin films deposited on GaAs (110) substrates. We show that a high contact resistance is associated with a low degree of crystallinity of Ti when deposited directly on GaAs (110), while Pt/Ti/Pt/Au electrical contacts results in a remarkable decrease of the contact resistance, which is attributed to the improvement of the metal layer quality observed in the first Pt/Ti layers deposited on GaAs (110) surface.

## Materials and Methods

### Nanowire Growth

The self-catalyzed Be-doped GaAs NWs were grown by solid-source molecular beam epitaxy (MBE) on lithography-free oxide patterns fabricated on p-Si (111) substrates by droplet epitaxy and spontaneous oxidation [32]. The NW growth temperature was 640 °C, as determined by pyrometer, and a Ga flux corresponding to planar 0.3 μm/h growth rate on GaAs (100) was used. A 60 s Ga wetting preceded the NW growth, which was initiated by providing As_2_ with V/III beam equivalent pressure ratio of 9 and Be flux corresponding to 2.0 × 10^19^ cm^−3^ p-type doping concentration; this was determined from the growth of planar Be-doped GaAs (100) calibration samples by room temperature Hall measurements. The growth duration was 60 min. More details of the growth methodology and the structural properties of the undoped and Be-doped NWs can be found in Refs. [11, 32, 33]. In short, the NWs are composed of pure zinc blende GaAs with the formation of a few twin planes [33]. The NWs have hexagonal shape with sidewalls composed exclusively from (110) oriented facets, as it was previously determined from structural analysis of the Be-doped NWs [33] and further confirmed in undoped GaAs NWs grown under similar conditions [12].

### Contacts Fabrication and Characterization

The NWs were mechanically transferred to a p-GaAs (100) substrate covered with a 200-nm-thick SiO_2_ layer, which was pre-patterned by photolithography and electron beam evaporation of Ti/Au pads for transport characterization. The position of the transferred wires on the substrate was identified by low magnification scanning electron microscopy (SEM) imaging. Positive electron beam resist was spin-coated on the substrate and exposed with electron beam on the electrical contact areas. The resist was developed in MIBK:IPA solution after electron beam exposure and possibly followed by an oxygen plasma treatment to remove the residual resist of the NW sidewalls, as described in Table 1. The effects of the oxygen plasma treatment on the device performance will be later discussed in the text. Prior to metal evaporation of the contact layers, the samples were chemically treated to remove the native oxide and passivate the exposed NW sidewalls, as described later in the text. The lift-off was done by dipping the sample in heated acetone, rinsing in IPA and blow drying with nitrogen.

**Table 1:** Fabrication details of electrical contacts used for transport characterization of Be-doped GaAs NWs

Process#	Oxygen plasma	Oxide removal	Surface passivation	Metal layers	RTA
P1	–	NH_4_OH:H_2_O	–	Ti/Pt/Au	–
P2	Yes	HCl:H_2_O	(NH_4_)_2_S_x_	Ti/Pt/Au	–
P3	–	HCl:H_2_O	(NH_4_)_2_S_x_	Ti/Pt/Au	–
P4	–	HCl:H_2_O	(NH_4_)_2_S_x_	Pt/Ti/Pt/Au	–
P5	–	HCl:H_2_O	(NH_4_)_2_S_x_	Pt/Ti/Pt/Au	400 °C, 30 s

We have developed five distinct processes combining different surface treatments of the exposed NW sidewalls with different metallic multilayers used as electrical contacts. This allowed us to determine the individual contributions of each parameter in the resulting contact resistance when applied to the p-type GaAs NWs. For the surface native oxide removal, we used either a 2.8% NH_4_OH or 3.7% HCl diluted in H_2_O followed by H_2_O rinsing. For the surface passivation, we used a 15% solution of (NH_4_)_2_S_x_ diluted in H_2_O (heated at 45 °C) followed by H_2_O rinsing. The metallic multilayer were deposited using e-beam evaporation and were either Ti/Pt/Au (20/20/200 nm) or Pt/Ti/Pt/Au (5/10/10/200 nm). A rapid thermal annealing (RTA) of 400 °C for 30 s was used for the Pt/Ti/Pt/Au multilayer. The processes used for each sample are specified in Table 1. For each sample, 4 evenly spaced electrical contacts were fabricated along the NW axis. In this work, we restrict the IV analysis to the contact pairs located in the center region of the NW. The IV data was obtained at room temperature using a Keysight probe station.

### Structural Investigation by Grazing Incidence X-ray Diffraction

In order to investigate the structural properties of the electrical contacts on the NWs, grazing incidence X-ray diffraction (GIXRD) patterns were measured from reference Ti, Pt, and Pt/Ti thin films evaporated on undoped GaAs (110) substrates. We prepared the thin film samples described in Table 2 using native oxide removal by HCl: H_2_O and surface passivation by (NH_4_)_2_S_x_ in the same way as for the NW devices. The small incidence angle of the X-rays used in GIXRD allows us to analyze metallic films with the same thickness as used in the NW contacts owing to the small penetration depth. The GIXRD patterns were measured using Cu Kα radiation with 1.54 Å wavelength and an incidence angle of *ω* = 0.75° in relation to the sample surface. The diffraction peak positions are indexed according to ICDD files #00-044-1294 and #00-004-0802 for hexagonal-close packed (HCP) Ti and face-centered cubic (FCC) Pt, respectively, and are corrected by accounting for the effect of refraction of the X-rays in GIXRD experiments as described in Ref. [34].

**Table 2:** Description of surface chemical treatments and metal layers deposited on GaAs (110) substrates for GIXRD analysis

Sample#	Oxide removal	Surface passivation	Metal layers
S1	HCl:H_2_O	–	Ti (20 nm)
S2	HCl:H_2_O	(NH_4_)_2_S_x_	Ti (20 nm)
S3	HCl:H_2_O	(NH_4_)_2_S_x_	Pt (5 nm)
S4	HCl:H_2_O	(NH_4_)_2_S_x_	Pt/Ti (5/20 nm)

## Results and Discussion

**Fig. 1:**
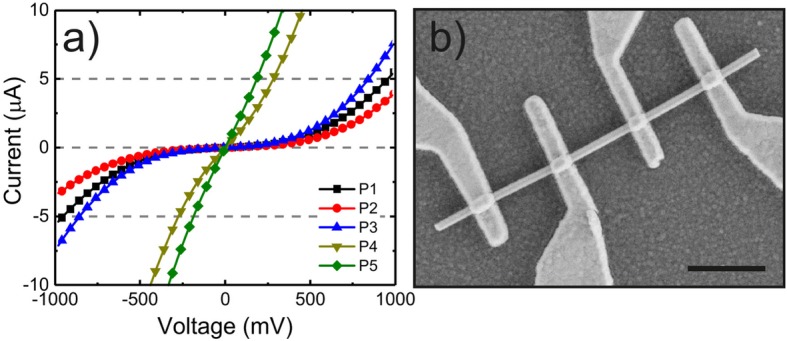
**a** IV from Be-doped GaAs NWs with electrical contacts fabricated using the processes P1–P5 as described in Table 1. **b** SEM image of a representative Be-doped GaAs NW with four evenly spaced electrical contacts. The scale bar is 1 μm

Figure 1a shows the IV characteristics for samples P1 to P5, and Fig. 1b an SEM image of a Be-doped GaAs NW with electrical contacts used for transport characterization. The almost symmetric, nonlinear shape of the IVs for P1 to P4 in Fig. 1a indicates that the contacts are of Schottky type with similar barrier heights for each contact [35]. The nonlinearity of the IV for sample P1 evidently shows that the standard p-GaAs process as in P1 does not yield Ohmic contacts as is the case for GaAs planar thin films. Usually, in doped GaAs NWs, HCl oxide removal is used, possibly followed by (NH_4_)_2_S_x_ surface passivation prior to metal evaporation for Ohmic contact formation [20, 21, 36, 37] instead of NH_4_OH. In addition, oxygen plasma treatment of the exposed NW surface has been previously used to remove the residual resist from the NW sidewalls [36, 38]. However, as a side effect, this process can induce surface defects in GaAs such as As vacancies, resulting in donor-like traps that are responsible for carrier compensation and therefore increasing the depletion layer width [5].

To evaluate the effect of the oxygen plasma treatment on the contact resistance of p-GaAs NWs, we compare the IV of a sample with (P2) and without (P3) oxygen plasma cleaning prior to the surface treatment by HCl and (NH_4_)_2_S_x_ in Fig. 1a. P2 yields the worst IV performance (defined as the electrical current value for the same applied voltage) in all samples but sample P3 by its turn exhibits better IV performance than the standard p-GaAs process P1, and the oxygen plasma cleaned P2. This implies two significant results: (i) the effect of the oxygen plasma treatment is detrimental on the contact resistance, and (ii) the P3 with HCl oxide removal combined with (NH_4_)_2_S_x_ surface passivation adds up to a lower Schottky barrier height of the metal-semiconductor interface compared to P1.

The IV performance and Ohmic character (evaluated qualitatively by the IV linearity) was strongly enhanced in P4 when compared to P3 by adding a 5-nm Pt layer under the Ti/Pt/Au multilayer, as can be seen from Fig. 1a. The contact resistance is further decreased in P5 after RTA 30 s at 400 °C, achieving a linear IV behavior and improved IV performance when compared to P4.

**Fig. 2:**
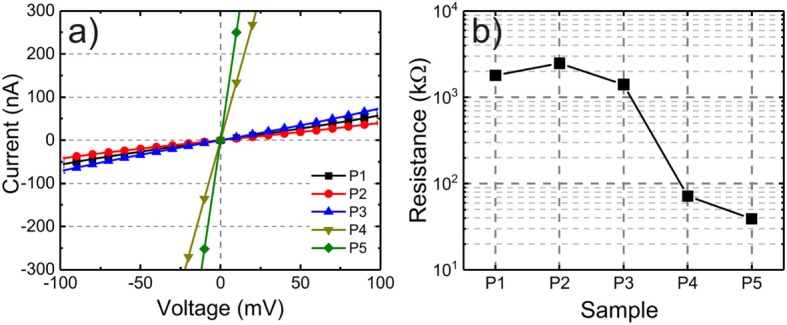
**a** IV from P1 to P5 in the 100 mV applied voltage range. **b** Total channel resistance of the P1–P5 obtained from linear fit of the IVs in **a**

In order to quantify the effect of the processing parameters on the contact resistance of samples P1–P5 (chemical treatments, metallic multilayer), we show in Fig. 2a the IVs from P1–P5 using a smaller bias range; in this case, the IVs exhibit linear behavior and are mainly governed by the contact resistance [35]. The total resistance from the channel (contacts + NW) in the 100 mV range from Fig. 2a was calculated from a linear fit of the IV characteristics curve, and the results are shown in Fig. 2b. Since the diameters of all investigated NWs are similar, and there is only small wire-to-wire variation in the dopant concentration, as we have previously reported [33], any changes of the total resistance were ascribed to the contact resistance. The higher resistance in P2 compared to P1 and P3 confirms the detrimental effect of oxygen plasma treatment from the qualitative analysis of the IVs in Fig. 1a. A remarkable result is the decrease in the total resistance from 1400 kΩ in P3 to 72 kΩ in P4 and a further decrease to 40 kΩ after RTA in P5, achieved by depositing an additional Pt layer prior to the Ti/Pt/Au multilayer used in samples P1–P3.

A more comprehensive understanding of the metal-semiconductor interface microstructure after the contact manufacturing is required to establish a correlation of the changes in the contact resistance observed in samples P1–P5. The use of Ti and Pt in Ohmic contact fabrication to GaAs has been previously reported [39, 40], and the structural properties of thin Ti and Pt films evaporated to GaAs (100) surface [41] and amorphous glass substrates [42, 43] have also been analyzed. However, no such detailed studies were found for GaAs (110) surface. The different surface orientation is expected to influence the crystallization dynamics of the Ti and Pt thin films. In addition, the surface chemical passivation by (NH_4_)_2_S_x_ could further influence the resulting thin films. The degree of crystallization of Pt (5 nm), Ti (20 nm), and Pt/Ti (5/20 nm) thin films deposited on undoped GaAs (110) substrate was investigated by GIXRD in order to obtain information of the structural properties of the first metallic layers in contact to the NWs in P1–P5. Prior to the metal evaporation, the GaAs (110) substrates went through the native oxide removal by HCl:H_2_O and the (NH_4_)_2_S_x_ surface passivation steps as the NW samples P3–P5. The details of the surface treatments and metallic thin films evaporated on GaAs (110) substrate are summarized in Table 2.

**Fig. 3:**
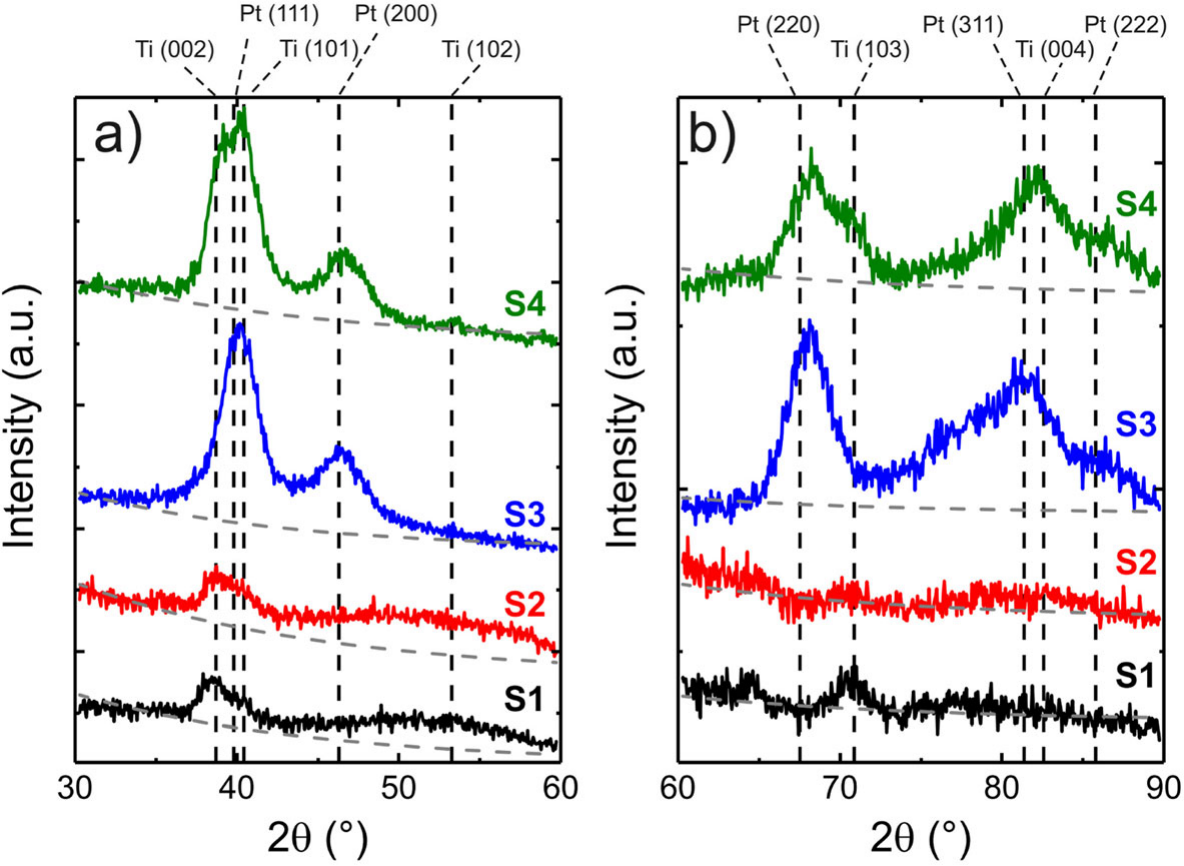
GIXRD patterns from samples S1–S4 of the **a** 30 to 60° diffraction angle range and (**b**) 60 to 90° range. The dashed gray lines represent exponential decay baselines that originate from diffuse X-ray scattering. The vertical dashed black lines correspond to the different diffraction planes of Ti and Pt, labeled at the top of **a** and **b**

The GIXRD patterns from samples S1–S4 are shown in the 30 to 60° diffraction angle range in Fig. 3a and in the 60 to 90° range in Fig. 3b. The diffraction patterns in Fig. 3 are vertically shifted and separated in two diffraction angle ranges to provide a better scaling for visualization. First, we focus on the effect of (NH_4_)_2_S_x_ surface passivation on the degree of crystallinity of Ti films evaporated on GaAs (110) substrate by comparing samples S1 and S2. In Fig. 3a, we observe overlapping low intensity Ti (002) and Ti (101) peaks centered at 38.4 and 40.2°, respectively, for both S1 and S2. Furthermore, a significantly broader Ti (102) peak centered at 53.0 ° is also observed for both samples, which suggests an amorphous character. The Ti (103) peak centered at 70.6° in Fig. 3b is only observed for S1, which is the only significant disparity between the samples. In general, the low intensities and broad peaks of S1 and S2 indicate a poor crystallinity of the Ti films when deposited on GaAs (110) surface after HCl oxide removal and regardless of the use of (NH_4_)_2_S_x_ passivation. In case of S3, for which Pt was deposited on GaAs (110) substrate with the same surface treatment as in S2, we observe much more pronounced Pt (111), Pt (200), Pt (220), Pt (311), and Pt (222) diffraction peaks centered at 39.8°, 46.3°, 67.5°, 81.3°, and 85.7°, respectively. This indicates that the Pt film in S3 exhibits a higher degree of crystallinity in comparison to the Ti samples. The same applies to S4 which shows similar Pt diffraction signatures as S3 in Fig. 3a, b. The broad and asymmetric peaks between 35°–45°, 65°–75°, and 75°–90° for S4 are formed due to the overlapping of Ti (002)-Ti (101)-Pt (111), Ti (103)-Pt (220), and Ti (004)-Pt (311)-Pt (222) diffraction peaks, respectively. A qualitative comparison of the GIXRD patterns from S2, S3, and S4 implies that the degree of crystallinity of Ti in S4 is at least on the same level as in S1. The Ti (103) peak at 70.6° is observed as a clear shoulder on the Pt (220) peak in Fig. 3b and the Ti (102) peak at 53.0° in Fig. 3a is present with low intensity but narrow linewidth in S4 while exhibiting a very broad, amorphous-like, peak in S1 and S2. This result suggests an improved degree of crystallinity of Ti when deposited on Pt instead of the GaAs (110) surface, which will in the following be directly correlated to the electrical contacts properties described in Figs. 1 and 2.

The GIXRD analysis of the metallic layers in S1–S4 allows us to correlate the degree of crystallinity of Ti and Pt deposited on GaAs (110) substrate and the total resistance results from P1–P5 in Fig. 2b. It is important to stress that in this work, we base our correlations of the changes in the total resistance of P1–P5 primarily on the GIXRD data obtained from S1–S4. We assume that other factors, such as the metal-NW interface quality due to the hexagonal geometry of the NWs sidewalls, have negligible contributions in the total resistance changes observed in P1–P5. The (NH_4_)_2_S_x_ surface passivation has a beneficial effect on the properties of the GaAs-metal interface as seem by comparing the IV and total resistance of samples P1 and P3, but with a low degree of crystallinity of the Ti film when deposited directly on GaAs (110) surface, as observed in S1 and S2. This could be the result of a reaction of sulfur with the overgrown Ti. In addition, it has been reported that Ti is highly reactive with the remaining impurities in the evaporation chamber during metal deposition [41], forming additional layers between the metal/GaAs and therefore increasing the contact resistance [5]. As previously discussed, the increase in contact resistance in P2 was ascribed to the possible surface damages caused by the oxygen plasma cleaning. The addition of a thin Pt layer between the Ti and GaAs (110) surface as in S4 results in a higher degree of crystallinity of the Ti film when compared to S1 and S2. This result can be correlated to the decrease of the total channel resistance from 1400 kΩ in P3 to 72 kΩ in P4 which is associated to a decrease of the contact resistance. The RTA further decreases the total channel resistance to 40 kΩ in P5 in addition to the increase of the Ohmic character of the IV shown in Fig. 2a. This result indicates that no detrimental reactions occur between Pt and GaAs surface in the annealing temperature and time used [29–31].

## Conclusions

The influence of surface chemical treatment prior to metal evaporation and the formation of Ohmic contacts to Be-doped self-catalyzed GaAs NWs was investigated by correlating transport characterization of single NWs and structural analysis of Ti, Pt, and Pt/Ti thin films deposited on GaAs substrates.We show that Ti exhibits low degree of crystallinity when deposited on GaAs (110) surface which directly affects the contact resistance of the NW devices. The deposition of a thin Pt layer on the NWs prior to Ti/Pt/Au results in a 95% decrease in the total electrical resistance of Be-doped GaAs NWs which is associated to the higher degree of crystallinity of Pt than Ti when deposited directly on GaAs (110). In addition, we show that thermal annealing of the metallic layers further decreases the contact resistance. These findings are of technological importance when designing Ohmic contacts to GaAs NWs-based devices and show the individual contributions of each processing step, described in Table 1, in the total resistance and Ohmic character of the NW devices. To further improve the device performance, a systematic optimization of the parameters of each individual step would be required. In particular, we show that the metal-semiconductor interface at the NW sidewalls plays a major role in the device performance and opens the way to further investigations of the crystallization process of metallic thin films deposited on different surface orientations of III–V semiconductor materials.

## Data Availability

The datasets supporting the conclusions of this study are included in within the article.

## Abbreviations

FCC: Face-centered cubic
GIXRD: Grazing incidence X-ray diffraction
HCP: Hexagonal-closed packed
IV: Current–voltage
NW: Nanowire
RTA: Rapid thermal annealing
SEM: Scanning electron microscopy
